# Supplementary figures and images for: Statin-mediated reduction in mitochondrial cholesterol primes an anti-inflammatory response in macrophages by upregulating Jmjd3
Source: eLife. 2024 Apr 11;13:e85964. doi: 10.7554/eLife.85964 (PMC11186637; doi:10.7554/eLife.85964)

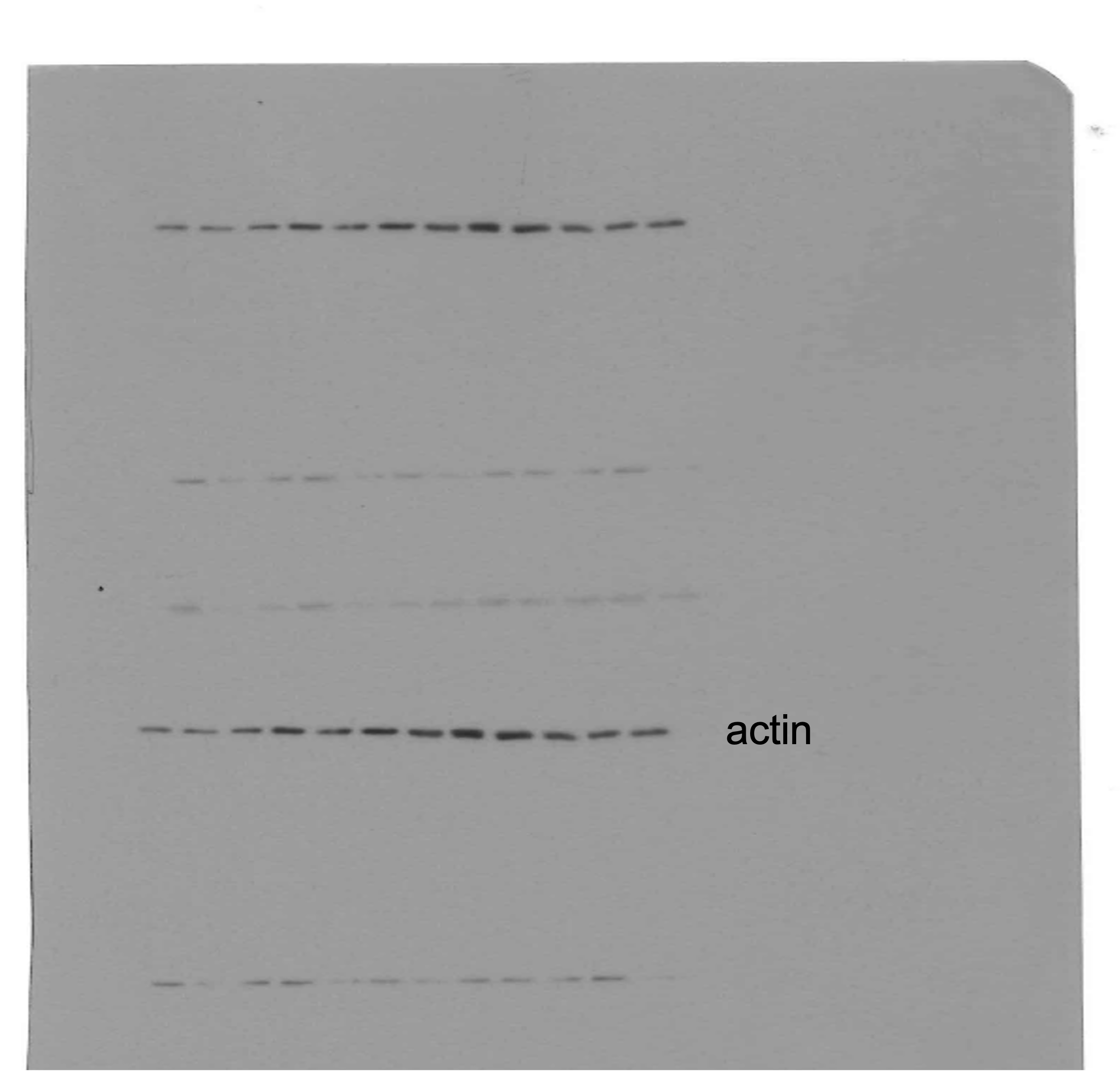

Supplement: Figure 1—source data 1. [file elife-85964-fig1-data1.zip › Fig1 C d source data/actin.png]

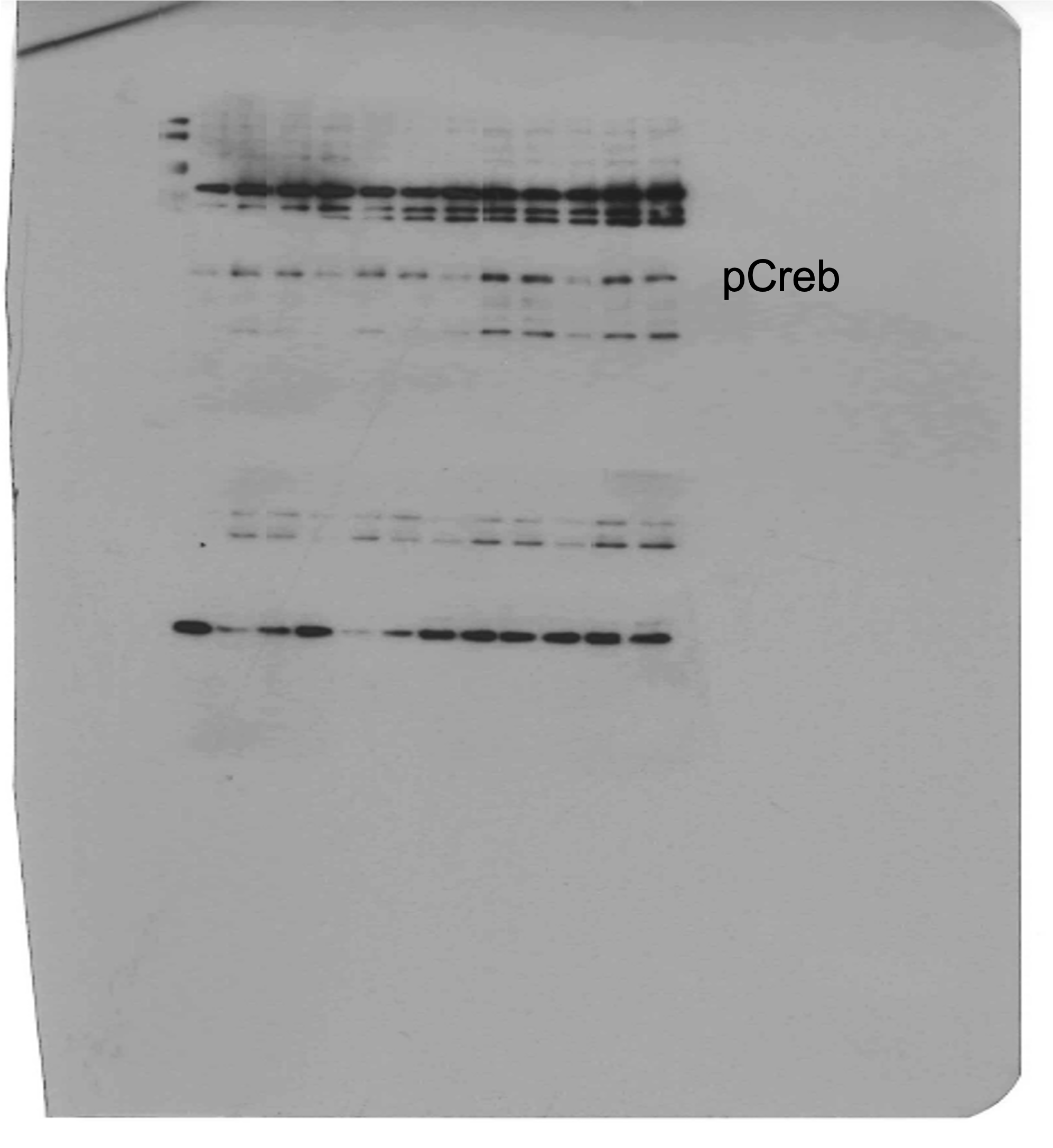

Supplement: Figure 1—source data 1. [file elife-85964-fig1-data1.zip › Fig1 C d source data/pCreb.png]

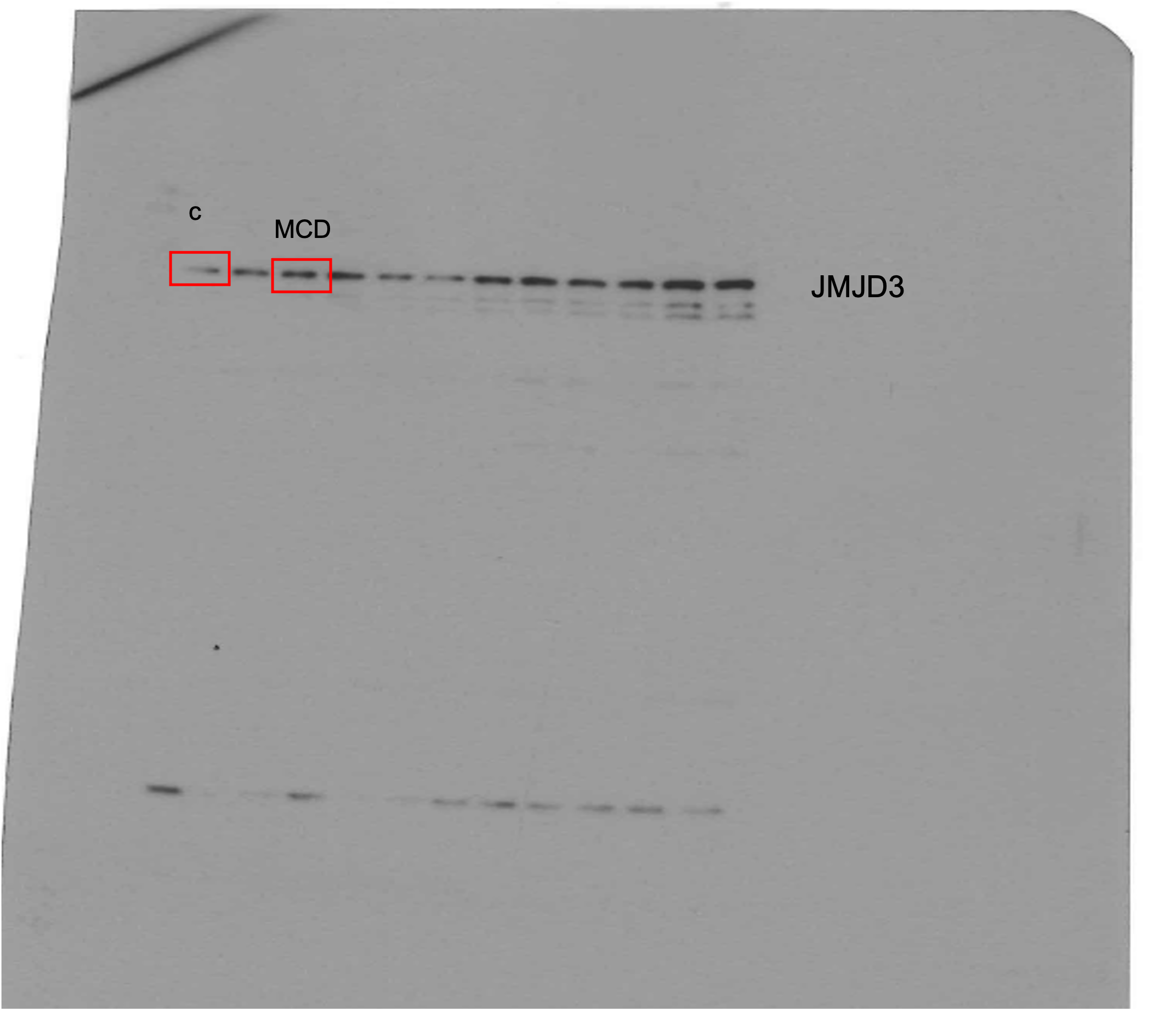

Supplement: Figure 1—source data 1. [file elife-85964-fig1-data1.zip › Fig1 C d source data/JMJD3 marked.png]

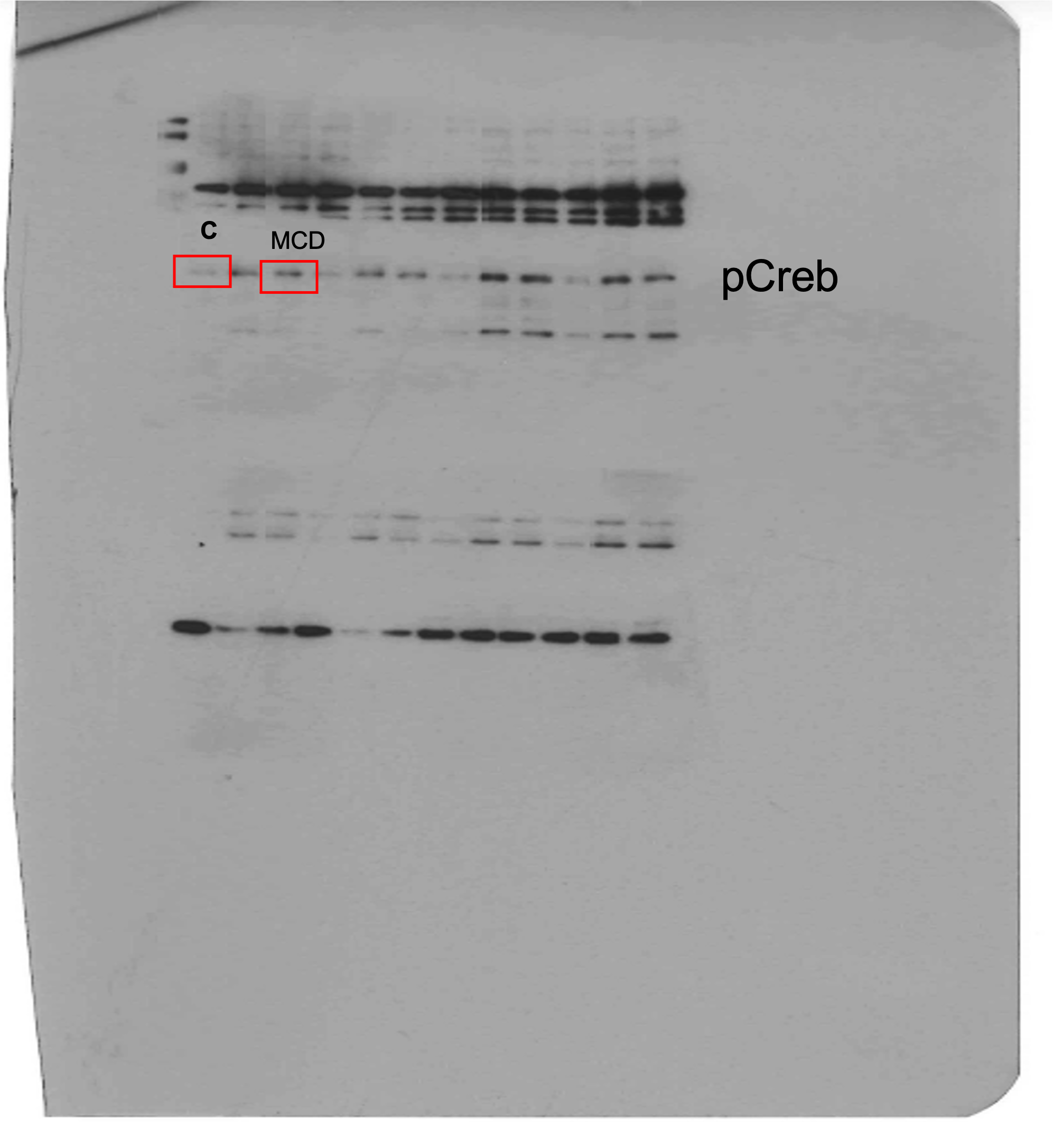

Supplement: Figure 1—source data 1. [file elife-85964-fig1-data1.zip › Fig1 C d source data/pCreb marked.png]

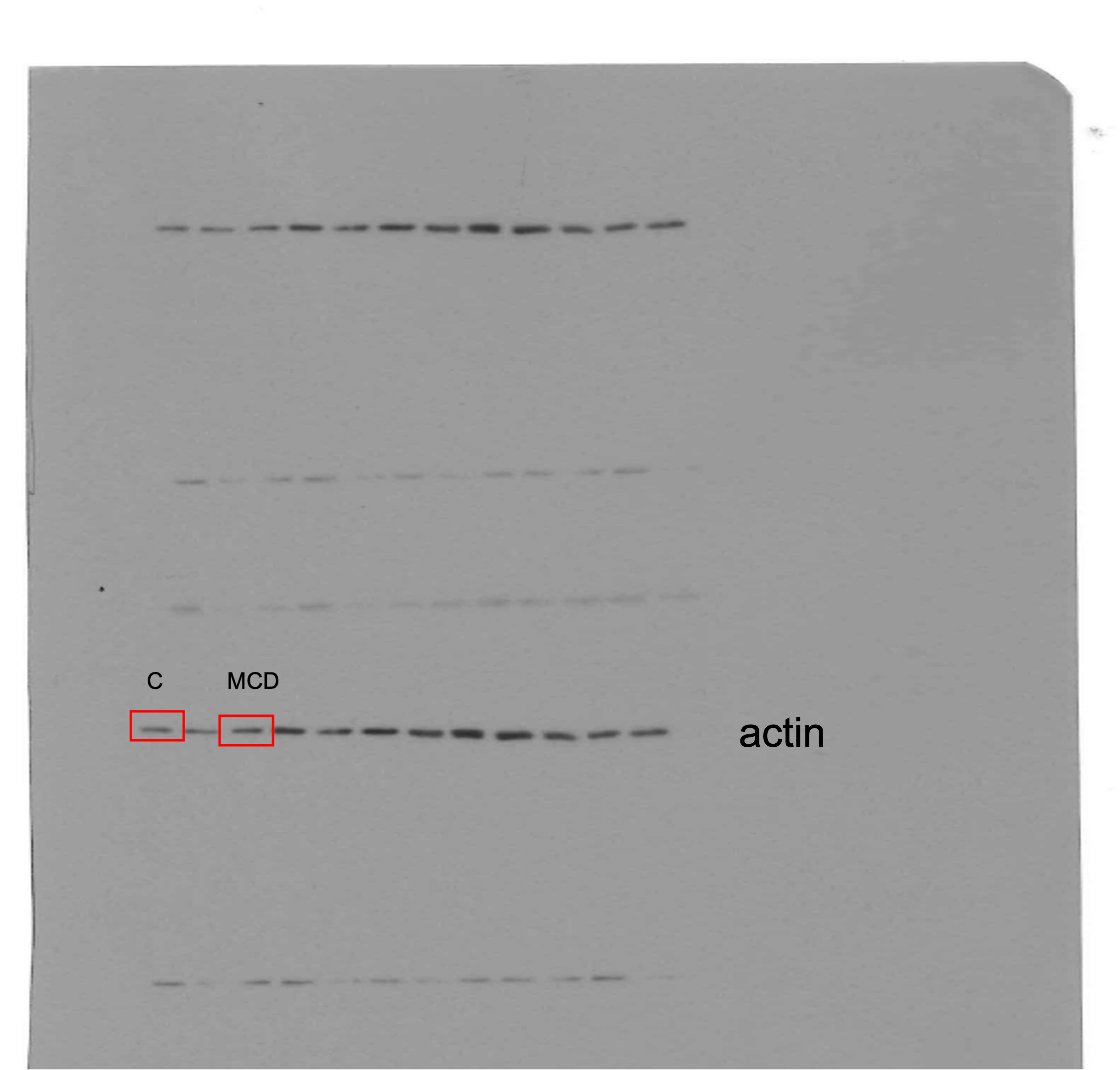

Supplement: Figure 1—source data 1. [file elife-85964-fig1-data1.zip › Fig1 C d source data/actin marked.png]

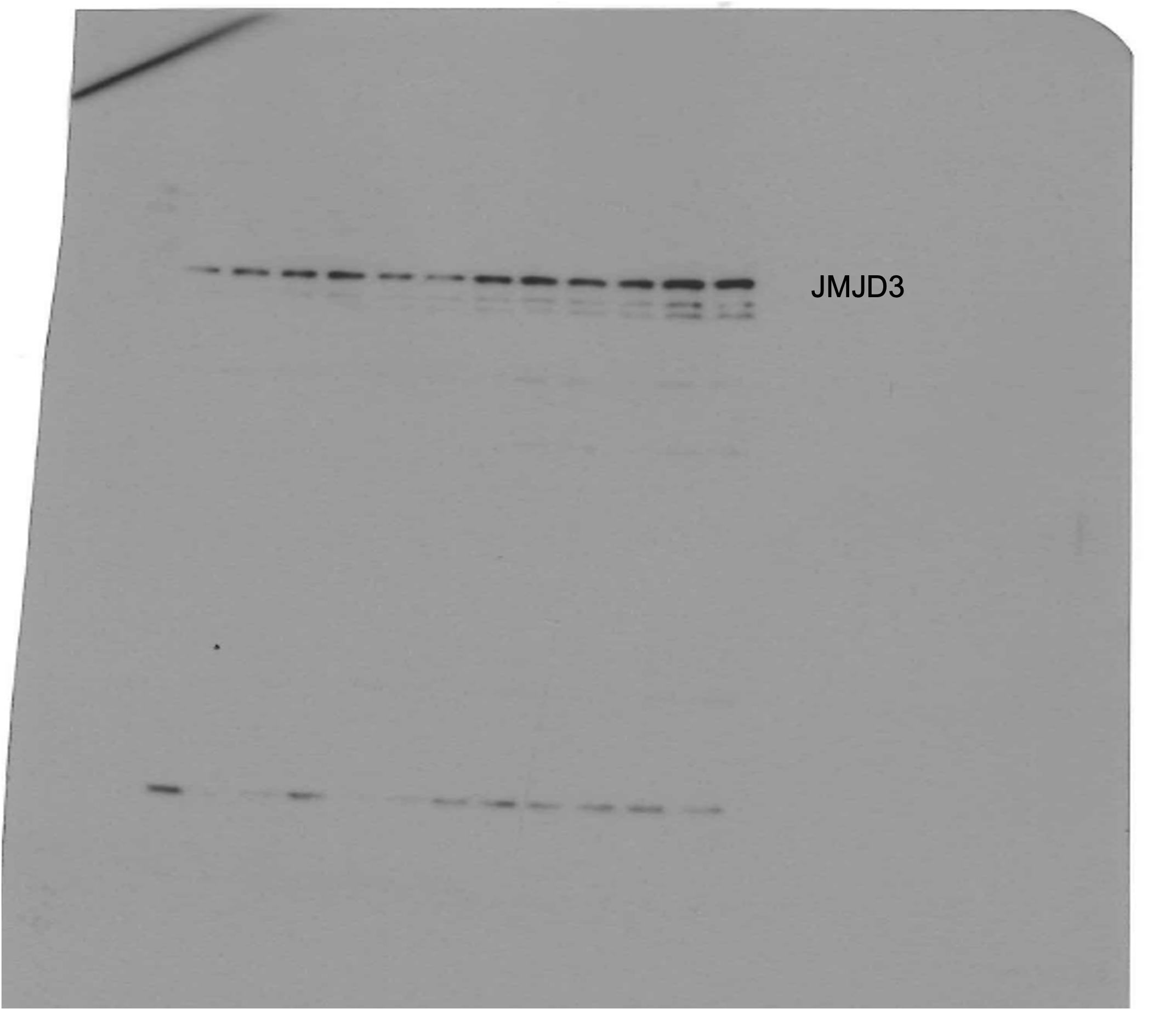

Supplement: Figure 1—source data 1. [file elife-85964-fig1-data1.zip › Fig1 C d source data/JMJD3.png]

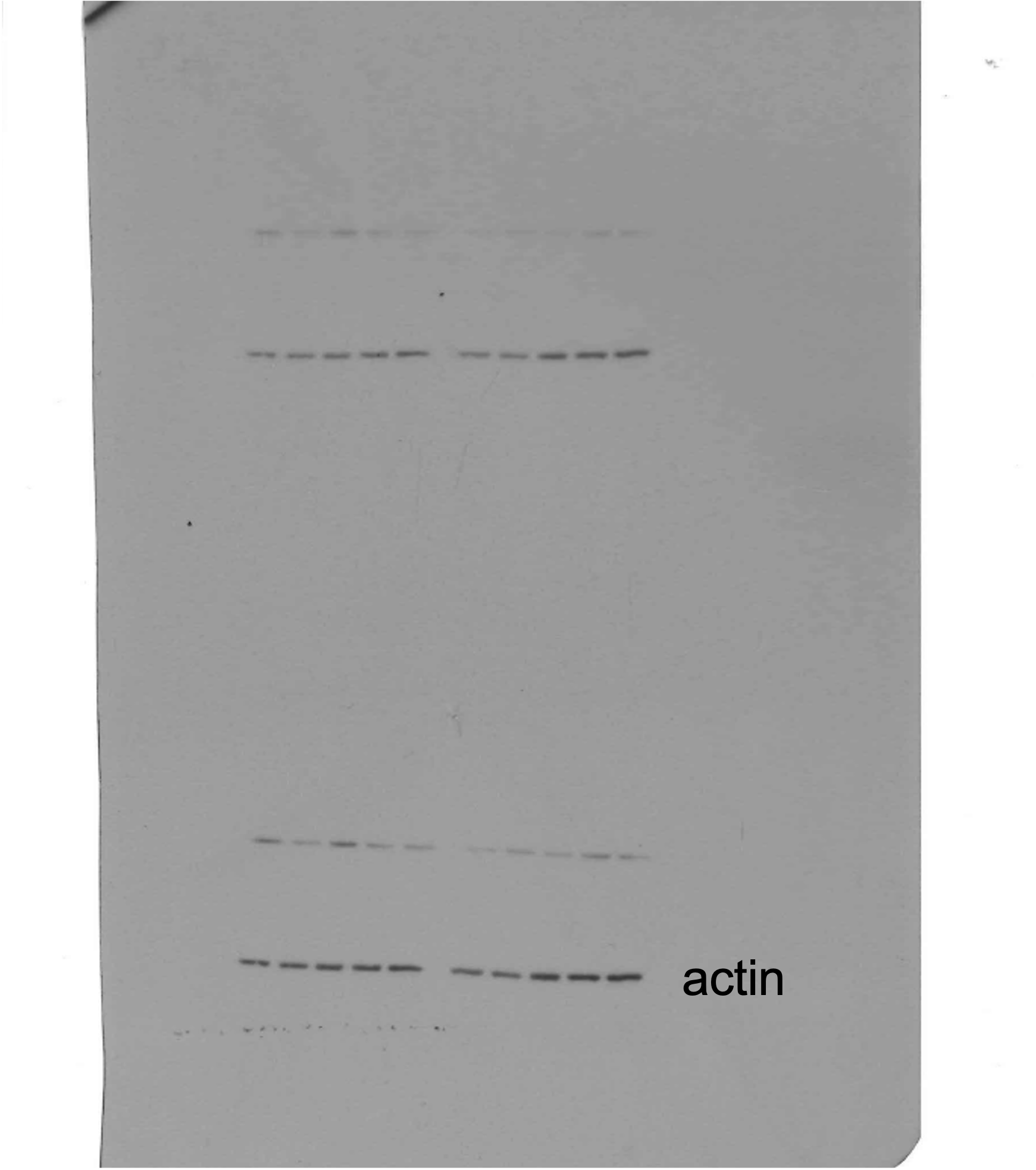

Supplement: Figure 1—source data 2. [file elife-85964-fig1-data2.zip › Fig1 C e source data/actin.png]

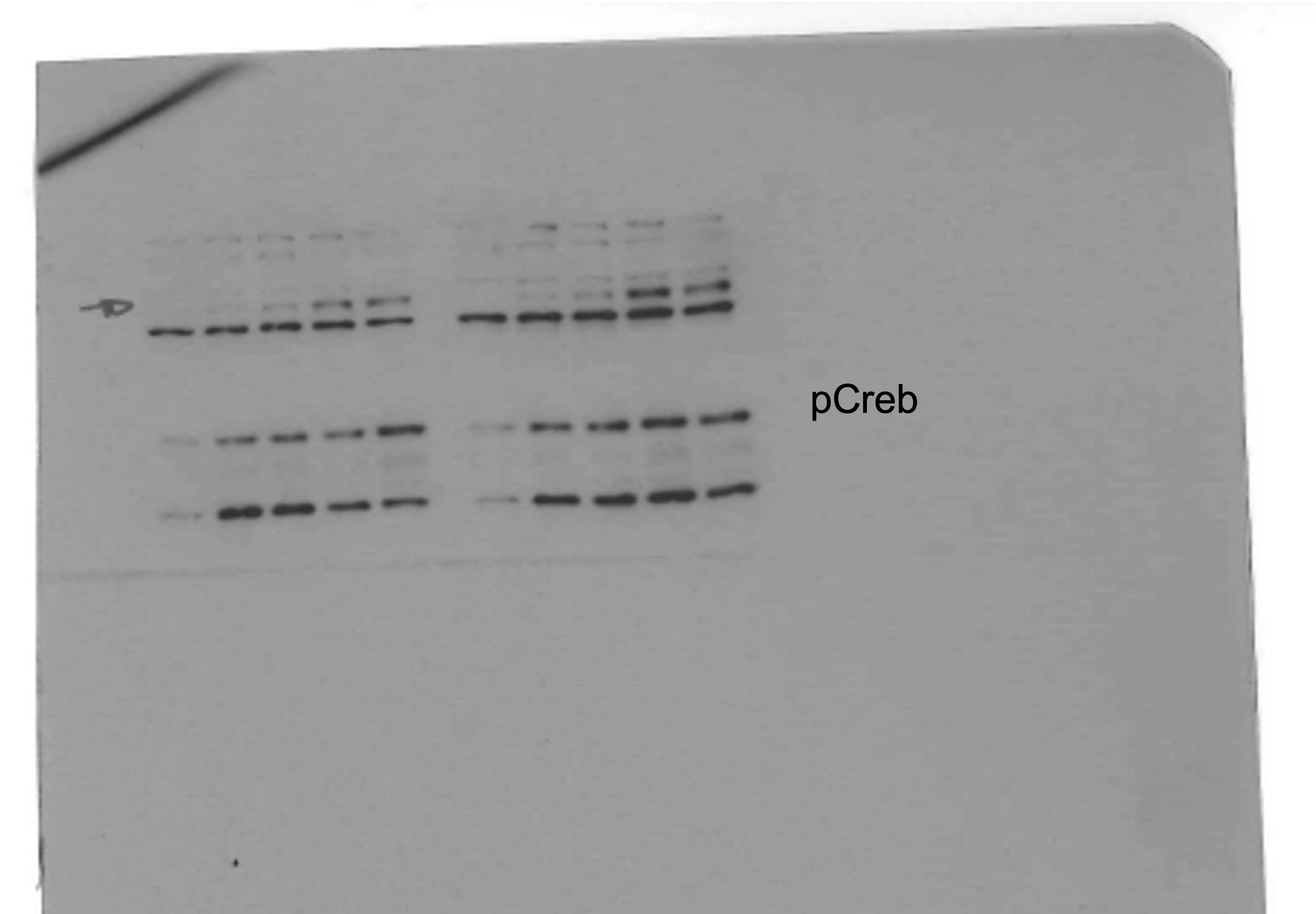

Supplement: Figure 1—source data 2. [file elife-85964-fig1-data2.zip › Fig1 C e source data/pCreb.png]

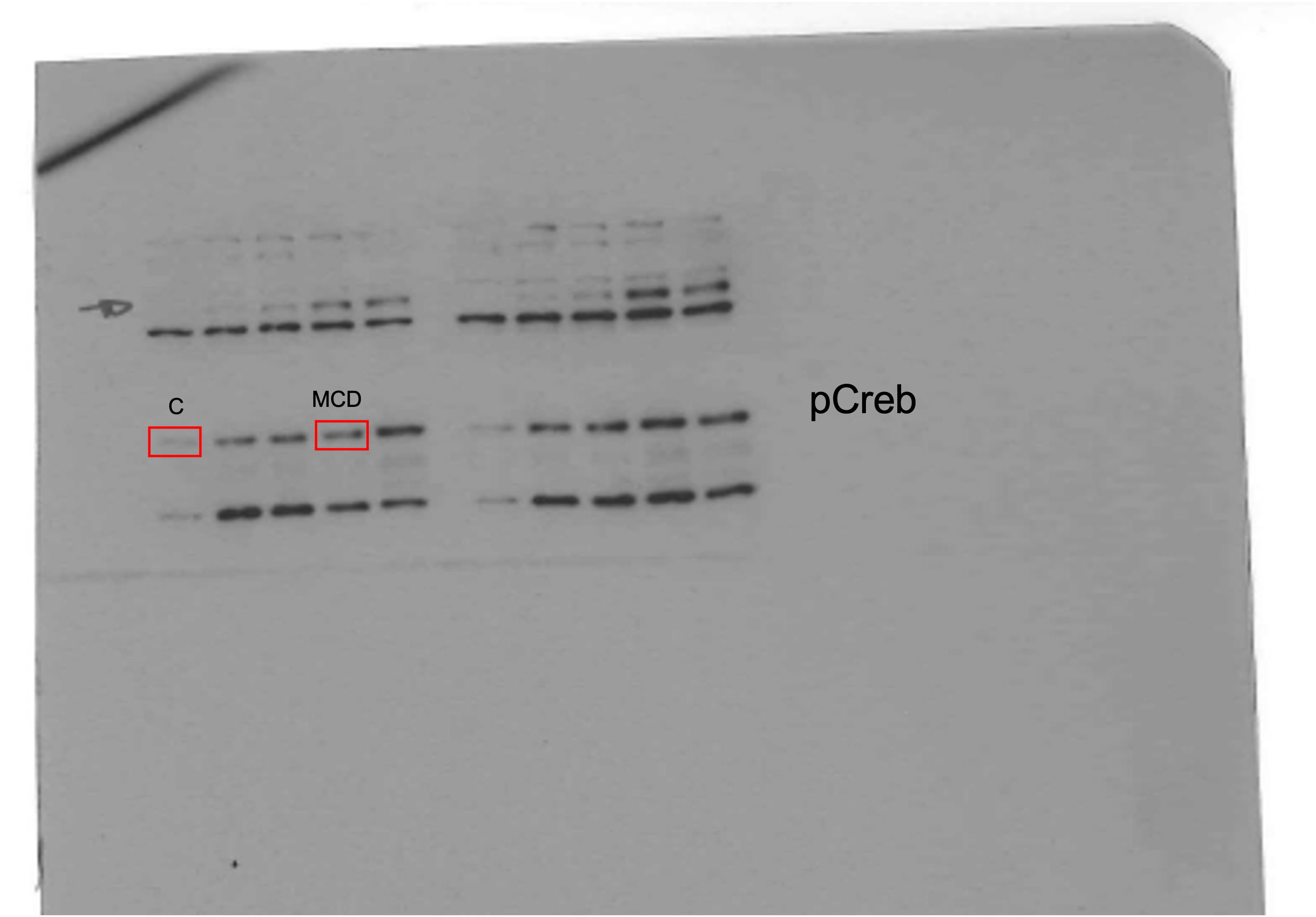

Supplement: Figure 1—source data 2. [file elife-85964-fig1-data2.zip › Fig1 C e source data/pCreb marked.png]

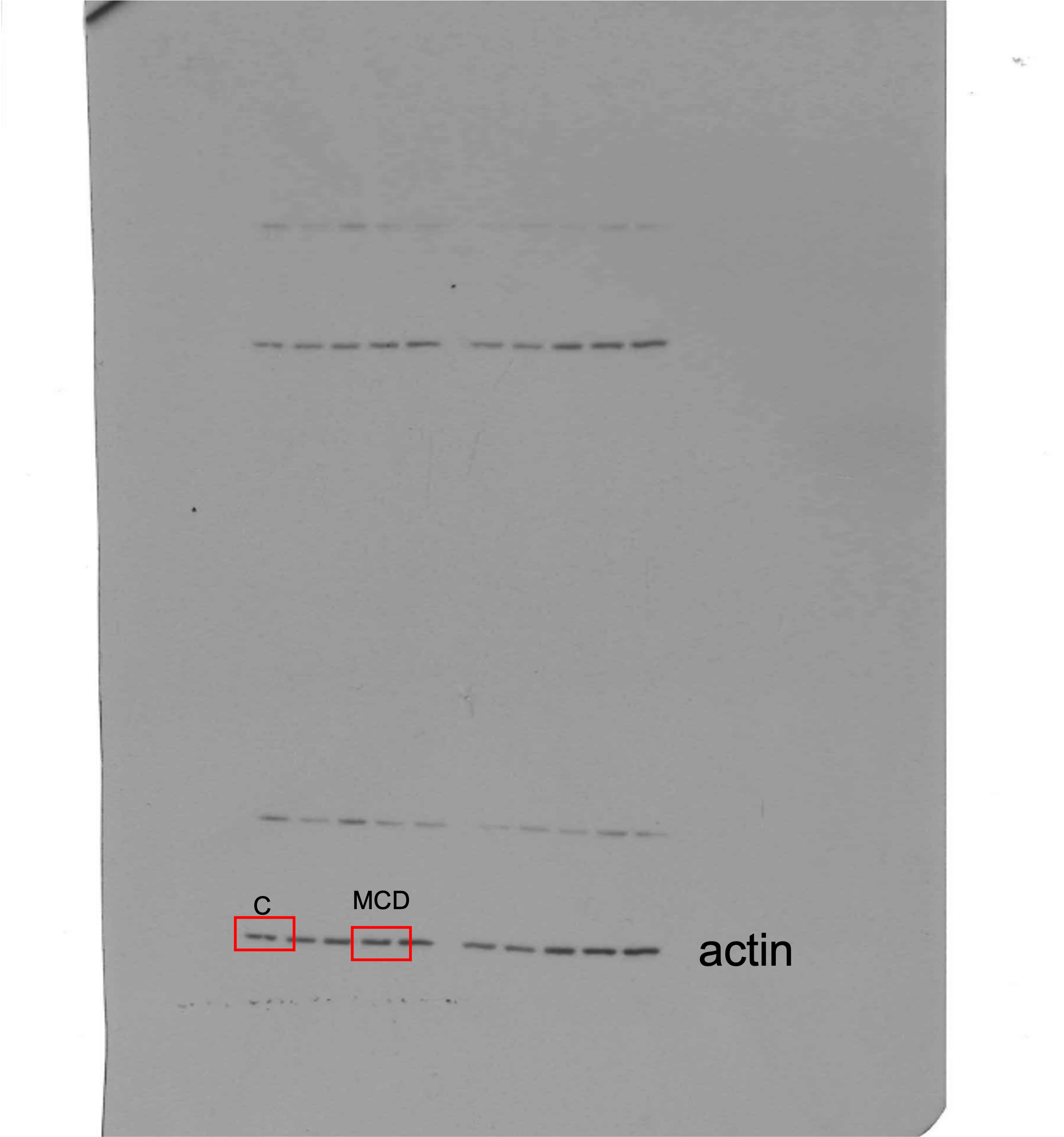

Supplement: Figure 1—source data 2. [file elife-85964-fig1-data2.zip › Fig1 C e source data/actin marked.png]

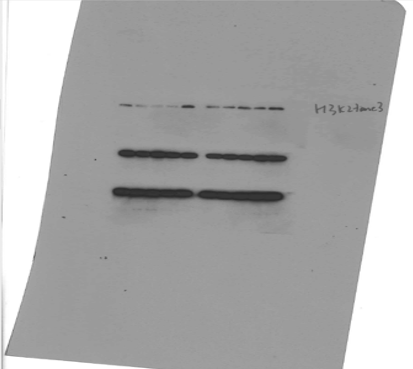

Supplement: Figure 1—source data 2. [file elife-85964-fig1-data2.zip › Fig1 C e source data/H3K27me3.png]

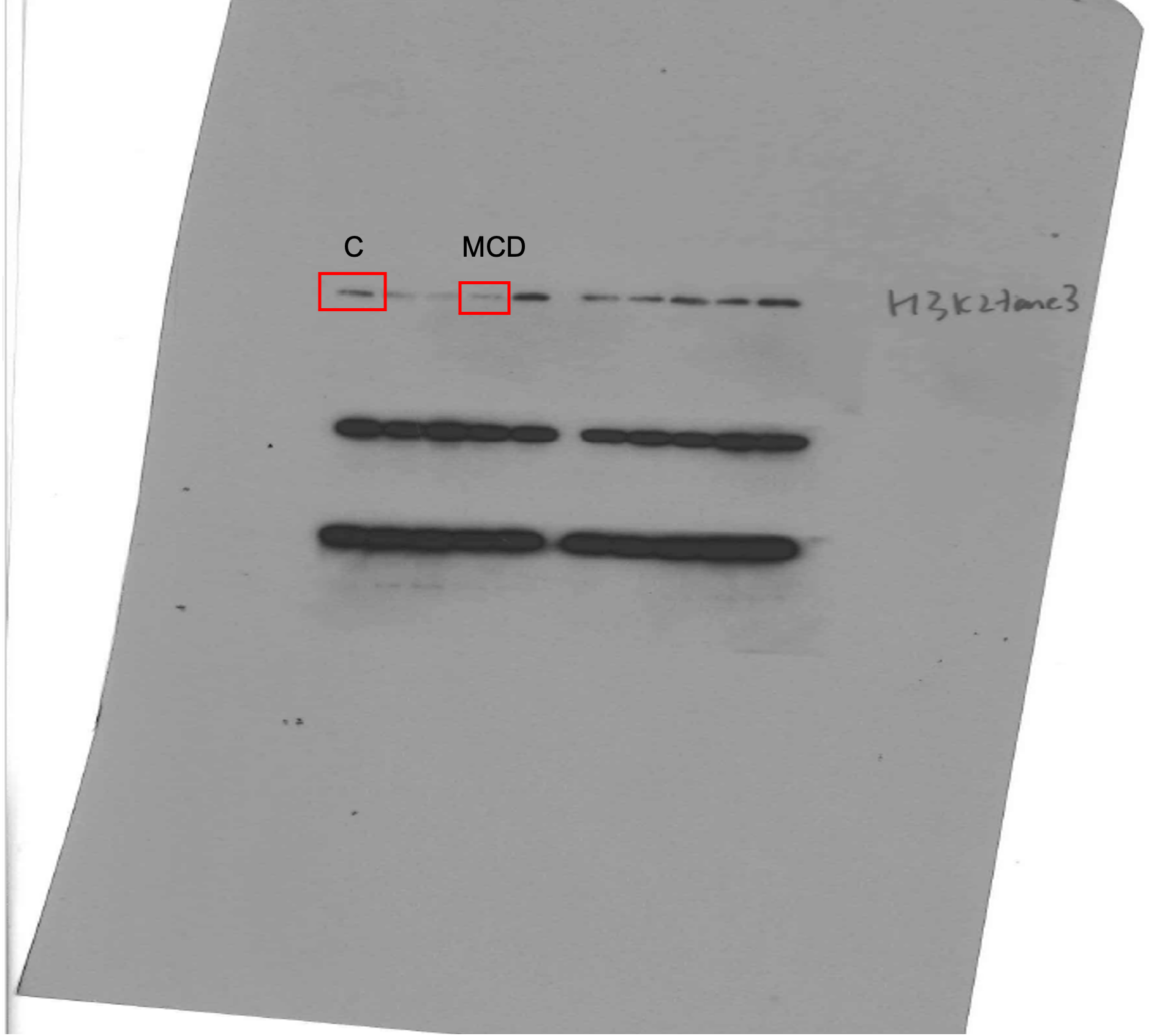

Supplement: Figure 1—source data 2. [file elife-85964-fig1-data2.zip › Fig1 C e source data/H3K27me3 marked.png]

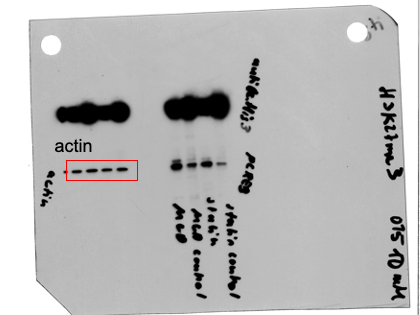

Supplement: Figure 1—figure supplement 3—source data 1. [file elife-85964-fig1-figsupp3-data1.zip › actin marked.png]

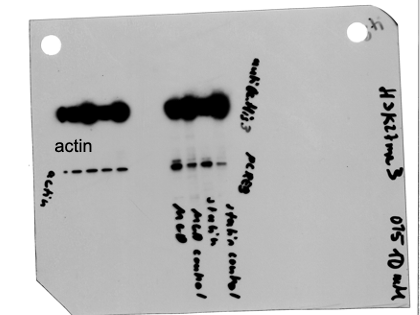

Supplement: Figure 1—figure supplement 3—source data 1. [file elife-85964-fig1-figsupp3-data1.zip › actin.png]

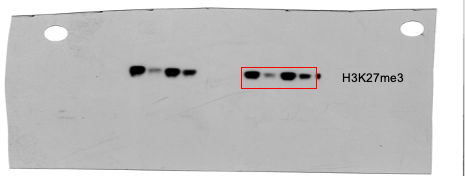

Supplement: Figure 1—figure supplement 3—source data 1. [file elife-85964-fig1-figsupp3-data1.zip › H3K27me3 marked.png]

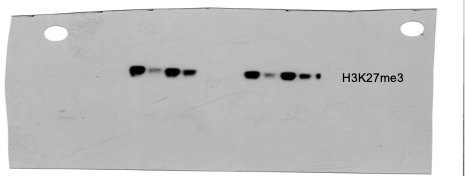

Supplement: Figure 1—figure supplement 3—source data 1. [file elife-85964-fig1-figsupp3-data1.zip › H3K27me3.png]

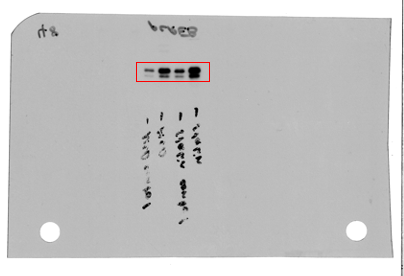

Supplement: Figure 1—figure supplement 3—source data 1. [file elife-85964-fig1-figsupp3-data1.zip › pCreb marked.png]

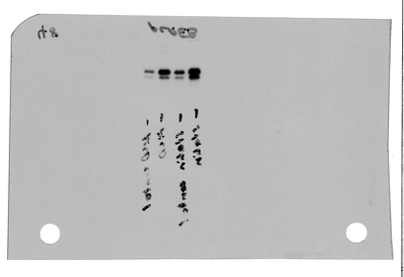

Supplement: Figure 1—figure supplement 3—source data 1. [file elife-85964-fig1-figsupp3-data1.zip › pCreb.png]
